# Supplementary material for: “I can’t make it safe, so I don’t do it”: Exploring obstetricians’ views on barriers and enablers to promoting vaginal birth after caesarean section in Bangladesh
Source: PLOS Glob Public Health. 2024 Dec 5;4(12):e0003963. doi: 10.1371/journal.pgph.0003963 (PMC11620460; doi:10.1371/journal.pgph.0003963)
Supplement: S1 File — (DOCX) [file pgph.0003963.s001.docx]

Topic guide

**Interview topic guide for obstetricians’ views on enablers and challenges to promoting Vaginal birth after caesarean section (VBAC) in Bangladesh**

**Introduction**

- Thank participant for making time for interview
- Introduction - Explain that the purpose of the interview is to collect the views and perspectives of obstetricians on the enablers and challenges to promoting VBAC in Bangladesh.
- Ensure each participant has read and understood the participant information sheet.
- Ensure key aspects from the information sheet are well-understood, primarily:

1. that the discussion will last around 30-40 minutes
2. that the content of the interview will remain confidential
3. that the participant’s name will not be used when reporting the findings
4. that any quotations used will be anonymised
5. that despite best efforts there is a chance that they may be identifiable from quotes should they express strong identifiable views.
6. a voice recorder will be used (phone), only to ensure that all the information from the interview is captured.

- Explain that the questions do not have a right or wrong answer, but you are interested in hearing their experiences and discussing their views and opinions.
- Confirm they are still happy to proceed.

**Profile of the participant**

- Job of the participant/ what stage of training are you at (if appropriate)
- Job history
  - Where did you train, have you worked anywhere other than Bangladesh
  - Private, public sectors, educational institutions
  - How many years of practice

**Questions**

Practice

- What are the **birth options** for women with previous CS?
- In your experience to what extent **is VBAC practiced** in Bangladesh?
  - Who - Certain health professionals
  - when/ why – under what circumstances is it performed/ recommended
  - where – certain facilities
- does practice of VBAC differ in **different settings** you have worked in?
  - Other countries or settings
  - Private vs public within Bangladesh
- Who are the people involved in the **decision-making process** regarding caesarean sections?
  - Obstetricians, mothers, family members
  - Is reason and timing documented.

Policy

- Are you aware of any **guidelines** that set out when VBAC should be performed?
  - Hospital guidelines, organisation guidelines, national guidelines?
  - If yes – what is the name/ where can I find them
  - If yes, are these policies put into practice?
- **Training** for obstetricians – is it mentioned/ covered

Barriers and facilitators to VBAC

- What **barriers** have you experienced regarding VBAC?
  - Environment
  - Staff – attitudes, training, experience
  - Barriers/ enablers to Decision-making?
  - neonatal outcomes, litigation etc.
  - Financial?
  - Perceptions of women and their family
- What about things that **facilitate** VBAC in Bangladesh?
- In your opinion, is the **level of practice of VBAC** in Bangladesh appropriate?
  - If YES – how can this be maintained
  - If no – why not, what could be done to promote this

**Additional information**

- Is there anything else you’d like to add?
- Are there any key documents you are aware of that you think would be relevant for this study?

**Closure**

- Provide a summary of that main points covered in the meeting
- Thank participants for their time and participation
