# Supplementary material for: “I can’t make it safe, so I don’t do it”: Exploring obstetricians’ views on barriers and enablers to promoting vaginal birth after caesarean section in Bangladesh
Source: PLOS Glob Public Health. 2024 Dec 5;4(12):e0003963. doi: 10.1371/journal.pgph.0003963 (PMC11620460; doi:10.1371/journal.pgph.0003963)
Supplement: Attachment — Submitted filename: Response to Reviewers.docx [file pgph.0003963.s003.docx]

Editorial team

PLOS Global Public Health

Thursday 29 August 2024

Dear Editor and Reviewer,

**RE: Revision of manuscript PGPH-D-24-00771**

**"I can't make it safe, so I don't do it": Exploring Obstetricians' views on barriers and enablers to promoting vaginal birth after Caesarean Section in Bangladesh**

Thank you for the opportunity to submit a revised version of this manuscript. We appreciate the insightful comments provided and have carefully considered each suggestion.

We have made several key revisions to the manuscript, in particular clarifying several aspects of the methodology to address previous ambiguity. All changes are highlighted in red on the manuscript using track changes for your convenience. Responses to each comment are provided below.

We appreciate your time and thoughtful review of the manuscript, which have significantly improved its quality. We also appreciate your recognition of the importance of our study’s results. We hope that these revisions address your concerns and will facilitate the paper’s publication, ensuring that these crucial findings are able to reach policymakers.

Kind regards,

Aimee Hairon

aimeehairon@gmail.com

In response to the comments and suggestions, the following changes have been made:

**Response to Editor comments:**

1. Please review your reference list to ensure that it is complete and correct. If you have cited papers that have been retracted, please include the rationale for doing so in the manuscript text, or remove these references and replace them with relevant current references. Any changes to the reference list should be mentioned in the rebuttal letter that accompanies your revised manuscript. If you need to cite a retracted article, indicate the article’s retracted status in the References list and also include a citation and full reference for the retraction notice

The reference list has been reviewed using EndNote20 and is correct and complete.

2. Please include a complete copy of PLOS’ questionnaire on inclusivity in global research in your revised manuscript.

Attached

3. In the online submission form, you indicated that "Excerpts of the transcripts relevant to the study are presented in the manuscript. Individual anonymised transcripts can be provided upon request".

All PLOS journals now require all data underlying the findings described in their manuscript to be freely available to other researchers, either

1. In a public repository, 
2. Within the manuscript itself, or 
3. Uploaded as supplementary information.

This policy applies to all data except where public deposition would breach compliance with the protocol approved by your research ethics board. If your data cannot be made publicly available for ethical or legal reasons (e.g., public availability would compromise patient privacy), please explain your reasons by return email and your exemption request will be escalated to the editor for approval. Your exemption request will be handled independently and will not hold up the peer review process, but will need to be resolved should your manuscript be accepted for publication. One of the Editorial team will then be in touch if there are any issues.

An exemption request email has been sent and passed on to the relevant team. The request was as follows:

“Point 3 of "Journal requirements" states that all data underlying findings need to be made freely available to other researchers. In the context of this study, this will not be possible as it would compromise participant anonymity and would also breach compliance with the protocol approved by the research ethics board.

Before agreeing to take part in the study, all participants read a participant information sheet and signed a consent form in which they agreed to the use of anonymised quotes for publication. They did not however consent to making interview transcripts freely available.

The subject of the interviews was potentially sensitive as it explored issues such as medical decision-making and health system issues, in the context of a hierarchal medical system. Many of the participants also worked together and knew each other. Therefore to allow participants to feel comfortable to express their own views without social desirability bias, ensuring confidentiality was a major priority during data collection. Making the transcripts freely available would compromise the anonymity of participants and breach ethical principles.

I would like to make an exemption request if possible. As indicated in the online submission form, individual anonymised transcripts can be provided upon request to the reviewer if necessary, but it will not be possible for these to be made freely available.”

**Response to the Reviewer’s comments:**

1. The objectives are aiming to generalize the findings to Bangladesh but the methodology does not allow such a generalization. Revision is recommended

Thank you for your comment, we agree with the reviewer and the phrasing of the aims and objectives has been revised accordingly.

2. The methodology section only states that a qualitative methodology will be used. However, the qualitative methodology is complex and a better description of what exact method and the philosophy behind the chosen methodology is required.

Thank you, more detail regarding the exact methodology and ideology behind it has been added to the study design section.

3. it appears that the researchers had decided on the sample size before the commencement of the study which is not a practice in qualitative research. However, reaching the point of saturation too is stated. Recommend editing the text appropriately.

Thank you for pointing this out, we agree that the original description of the recruitment process was somewhat ambiguous. The sampling and recruitment section has been edited to provide better clarification.

4. Under participant selection, the authors have selected participants from different categories. However, it is not clear if there was saturation for each of these categories. Different categories may have different ideas and knowledge which may not be reflected in the interviews with other categories.

Thank you for your comment, the reasoning behind the use of different sampling categories and how it was used in practice have been added to the sampling and recruitment section.

5. At the end of the data collection section, authors refer to experts in the subject who interviewed for triangulation purposes. However, no information on why they are considered as experts and how they were selected is provided.

Thank you for highlighting this. While it is important we maintain confidentiality and therefore cannot specify which organisations these individuals are affiliated with, more detail about how these people were selected and why they were felt to be “experts” has been added.

6. The authors conclusions are for the whole of Bangladesh, however qualitative studies do not allow such generalizations.

Thank you for pointing this out, we agree that qualitative studies have limitations in terms of generalizability. To address this concern, we have revised the phrasing of the conclusion to be more specific about the study population.
