# Supplementary material for: “I can’t make it safe, so I don’t do it”: Exploring obstetricians’ views on barriers and enablers to promoting vaginal birth after caesarean section in Bangladesh
Source: PLOS Glob Public Health. 2024 Dec 5;4(12):e0003963. doi: 10.1371/journal.pgph.0003963 (PMC11620460; doi:10.1371/journal.pgph.0003963)
Supplement: Attachment — Submitted filename: Response to Reviewers.docx [file pgph.0003963.s004.docx]

Editorial team

PLOS Global Public Health

Wednesday 23^rd^ October 2024

Dear Editor and Reviewer,

**RE: Revision of manuscript PGPH-D-24-00771R1**

**"I can't make it safe, so I don't do it": Exploring Obstetricians' views on barriers and enablers to promoting vaginal birth after Caesarean Section in Bangladesh**

Thank you for the opportunity to submit a revised version of this manuscript. We have viewed your suggestions and made the necessary changes. All changes are highlighted in red on the manuscript using track changes for your convenience. Responses to each comment are provided below.

We appreciate your time and thoughtful review of the manuscript, which has significantly improved the manuscript and helped ensure these important findings are communicated effectively. We hope that these revisions address your concerns and will facilitate the paper’s publication, ensuring that these crucial findings are able to reach policymakers.

Kind regards,

Aimee Hairon

aimeehairon@gmail.com

In response to the comments and suggestions, the following changes have been made:

**Journal Requirements:**

1. Please review your reference list to ensure that it is complete and correct. If you have cited papers that have been retracted, please include the rationale for doing so in the manuscript text, or remove these references and replace them with relevant current references. Any changes to the reference list should be mentioned in the rebuttal letter that accompanies your revised manuscript. If you need to cite a retracted article, indicate the article’s retracted status in the References list and also include a citation and full reference for the retraction notice.

The reference list has been reviewed using EndNote20 and is correct and complete.

**Reviewers' comments:**

6. Review Comments to the Author

Reviewer #2: Great revisions, thank you! One minor comment might be to add sections to your result section by theme. Extremely minor but it might help to break up with results section.

Thank you for this comment. In the previously submitted manuscript, the results section was indeed subdivided into the categories and themes identified in Figure 1. However, we appreciate that the formatting of headings and subheadings may not have been entirely clear. The manuscript has now been edited to ensure that the sections are more distinct for better readability.
